# Primary adrenal Ewing’s sarcoma family of tumors with tumor thrombus of the inferior vena cava: a case report

**DOI:** 10.1186/s13256-023-03837-w

**Published:** 2023-03-24

**Authors:** Kaya Atagi, Takashi Karashima, Keisuke Mizutani, Hideo Fukuhara, Satoshi Fukata, Yujiro Miura, Atsuyuki Mitsuishi, Kazuhiro Hanazaki, Sunao Uemura, Ryohei Miyazaki, Takashi Anayama, Mayuka Yamane, Mizu Sakai, Mitsuko Iguchi, Kenji Yorita, Keiji Inoue

**Affiliations:** 1 Department of Urology, Kochi Medical School, Nankoku, 783-8505 Japan; 2 Department of Cardiovascular Surgery, Kochi Medical School, Nankoku, 783-8505 Japan; 3 Department of Digestive Surgery, Kochi Medical School, Nankoku, 783-8505 Japan; 4 Department of Respirator Surgery, Kochi Medical School, Nankoku, 783-8505 Japan; 5 Department of Respiratory Medicine, Kochi Medical School, Nankoku, 783-8505 Japan; 6 Laboratory of Diagnostic Pathology, Kochi Medical School Hospital, Nankoku, 783-8505 Japan; 7 Department of Diagnostic Pathology, Japanese Red Cross Kochi Hospital, Kochi, 780-8562 Japan; 8 Department of Urology, Kochi Medical School, Kochi University, Kohasu, Oko, Nankoku, Kochi 783-8505 Japan

**Keywords:** Ewing’s sarcoma, Adrenal gland, Tumor thrombus, Vena cava

## Abstract

**Background:**

Ewing’s sarcoma is a malignant neoplasm that mainly occurs in skeletal tissue but can rarely arise in soft tissues. Recently, small round cell tumors (including Ewing’s sarcoma) caused by chromosomal translocations have been collectively termed Ewing’s sarcoma family of tumors. We report a rare case of primary adrenal Ewing’s sarcoma family of tumors with tumor thrombus.

**Case presentation:**

A 22-year-old Asian woman was referred to our hospital with a left retroperitoneal tumor 19 cm in diameter. Tumor thrombus was identified from the left adrenal vein to the inferior vena cava, infiltrating the right atrium. Total tumor excision with left adrenalectomy, nephrectomy, and thrombectomy was performed under hypothermic circulatory arrest, followed by seven courses of adjuvant chemotherapy. The patient has shown no signs of recurrence as of 26 months postoperatively.

**Conclusion:**

Radical surgery combined with systemic chemotherapy may contribute to good prognosis in patients with primary adrenal Ewing’s sarcoma family of tumors.

## Introduction

Ewing’s sarcoma (ES) is a malignant neoplasm that occurs primarily in the skeletal tissues of the long bones and pelvis in children and young adults, but on rare occasions arises in soft tissues [1]. Recently, small round cell tumors caused by chromosomal translocations have been collectively termed Ewing’s sarcoma family of tumors (ESFT) [2]. We report herein a patient with ESFT arising in the left adrenal gland who achieved complete remission and long-term survival after surgery and adjuvant chemotherapy.

## Case presentation

**Fig. 1:**
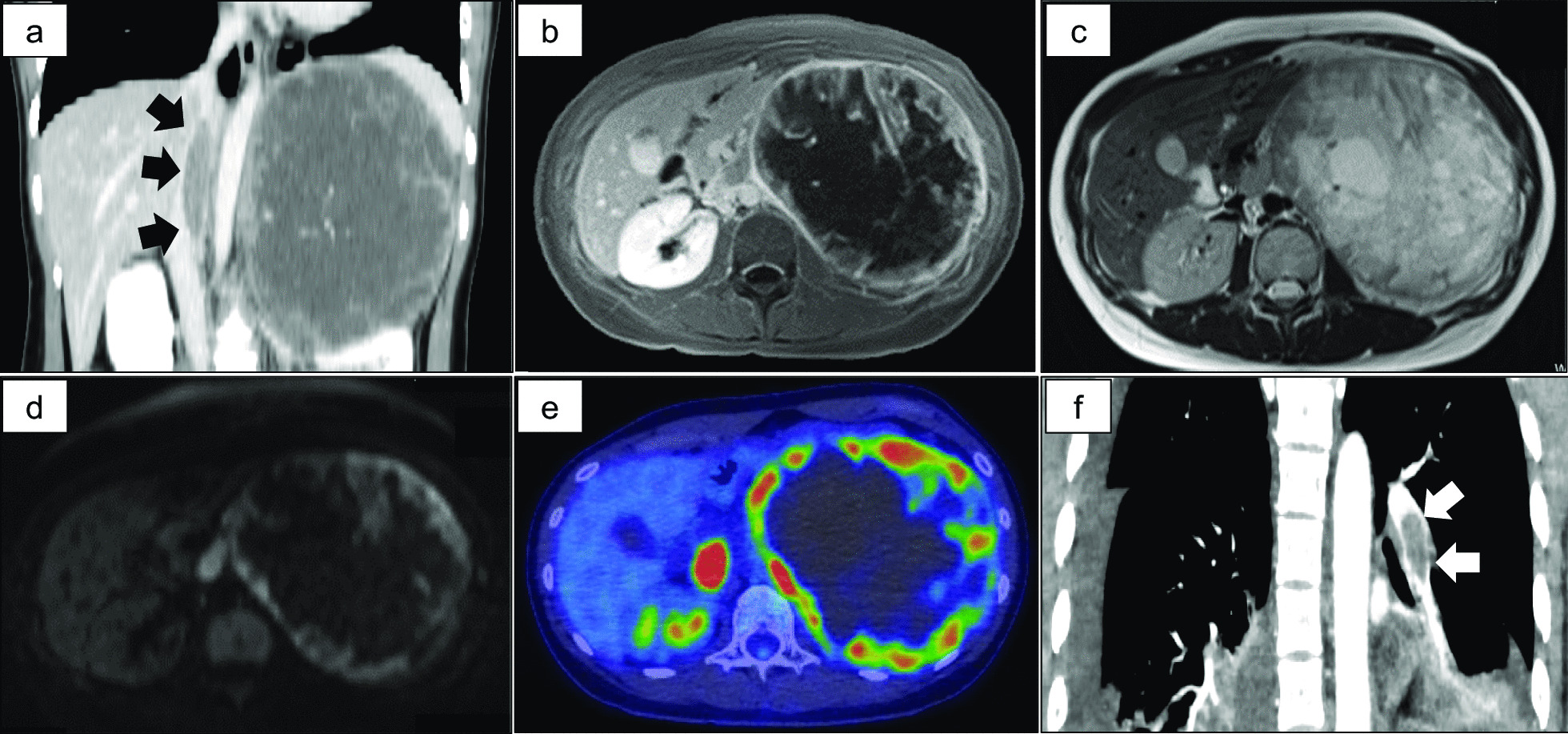
Preoperative CT, MRI, and FDG-PET. **a** Contrast-enhanced CT reveals irregular contrast in a left retroperitoneal tumor 19 cm in diameter, showing tumor thrombus in the left renal vein, adrenal vein, and vena cava. Black arrows indicate tumor thrombus within the inferior vena cava. **b**–**d** MRI reveals an encapsulated multilocular tumor with partially solid component, showing hypointensity on T1-weighted imaging, irregular hyperintensity with a septum on T2-weighted imaging, and strong signals on DWI. **e** On ^18^F-FDG-PET–CT, maximum standardized uptake value is 12.2 and no distant metastases are evident. **f** Contrast-enhanced thoracic CT on day 4 after surgery reveals tumor embolization in the left pulmonary artery (white arrow)

A 22-year-old nulligravid Asian woman consulted a local clinic with a chief complaint of left thoraco-abdominal pain for 2 months. She was introduced to our hospital after diagnostic imaging revealed a large retroperitoneal tumor with tumor thrombus of the inferior vena cava. She did not have any complications or relevant past or family history. Results from blood testing, including tumor markers and hormonal examinations, and urine testing were all within normal ranges. Contrast-enhanced computed tomography (CT) revealed irregular contrast enhancement of a left retroperitoneal tumor measuring 19 cm in diameter, along with tumor thrombus in the left adrenal and renal veins and inferior vena cava (Fig. 1a). Magnetic resonance imaging (MRI) revealed an encapsulated multilocular tumor with a partially solid component, showing hypointensity on T1-weighted imaging, irregular hyperintensity with septa on T2-weighted imaging, and strong signals on diffusion-weighted imaging (DWI) (Fig. 1b–d). Strong accumulation was seen on ^18^F-fluorodeoxyglucose-positron emission tomography (FDG-PET), with a maximum standardized uptake value of 12.2 and no distant metastases (Fig. 1e). Preoperative transthoraco-abdominal and intraoperative transesophageal ultrasonography revealed tumor thrombus in the vena cava extending into the right atrium (data not shown). The patient underwent en bloc tumorectomy with left adrenalectomy and nephrectomy, and thrombectomy by open-heart and open-abdominal surgery. The right atrial tumor thrombus was excised from the distal side via both vena caval and right atrial approaches under complete circulatory arrest as a deep hypothermic procedure without distal clamping of the vena cava. On day 4 after surgery, a tumor embolism that might have migrated intraoperatively was found in the left pulmonary artery. The tumor was urgently resected by left thoracotomy (Fig. 1f).

**Fig. 2:**
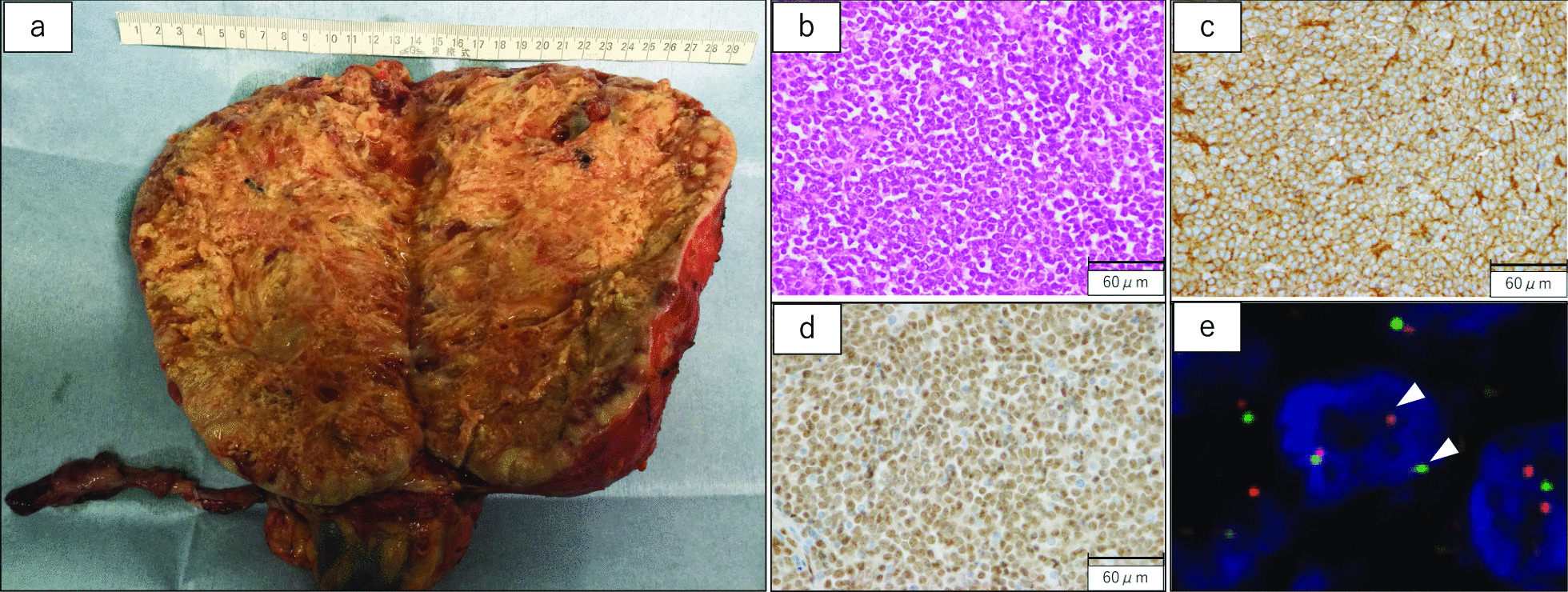
Macro- and microscopic findings of the tumor, and FISH assay of tumor cells. **a** The tumor cross-section is grayish-white in color with internal necrosis. **b** Microscopic examination with hematoxylin and eosin staining reveals uniform small round cells with round nuclei, intense chromatin staining, and a high nucleus-to-cytoplasm ratio proliferating solidly and partially showing a rosette-like structure. Immunohistochemical examination with anti-CD99 (**c**) and FLI-1 (**d**) antibodies shows strongly positive results. **e** A typical result of FISH assay to identify the chromosomal break point of EWSR1 in paraffin-embedded tissue reveals break-apart signals, shown as separate red and green signals (white arrowheads)

Macroscopic examination revealed a tumor measuring 19 × 14 × 8 cm. The cross-sectional surface was grayish white in color with internal necrosis (Fig. 2a). Microscopic findings for the tumor using hematoxylin and eosin (HE) staining included uniform small, round cells with round nuclei, intense chromatin staining, and a high nucleus/cytoplasm ratio proliferating solidly and showing a partially rosette-like structure. Frequent mitosis was evident, with a count of 32 per 10 high-power fields. Small cells resembling the existing adrenal cortex were occasionally observed in the region just below the capsule, so the tumor was considered to have been primarily derived from the adrenal gland (Fig. 2b). Immunohistochemical examination of the tumor with anti-cluster of differentiation (CD)99 and Friend leukemia integration 1 transcription factor (FLI-1) antibodies yielded strongly positive results (Fig. 2c, d), but results for CD34, leukocyte common antigen, terminal deoxynucleotidyl transferase, chromogranin A, and desmin were negative (data not shown). We performed a dual-color, break-apart fluorescence *in situ* hybridization (FISH) assay to identify the chromosomal break point of Ewing’s sarcoma breakpoint region 1 (EWSR1) in paraffin-embedded tissue (Abbott Molecular Inc., Des Plaines, IL, USA). EWSR1 in this case showed gene splitting in 87% of cells. Typical EWSR1 break-apart signals are shown in Fig. 2e. These findings led to a final diagnosis of ESFT.

Seven cycles of traditional alternating adjuvant chemotherapy with vincristine (1.5 mg/m^2^ on day 1), doxorubicin (37.5 mg/m^2^ on days 1 and 2), cyclophosphamide (1200 mg/m^2^ on day 1), ifosfamide (1800 mg/m^2^ on days 1–5), and etoposide (100 mg/m^2^ on days 1–5) (VDC-IE) were administered for 7 months from 73 days after surgery. Before commencing chemotherapy, oocytes were conserved. Grade 4 neutropenia resolved with administration of granulocyte colony-stimulating factor and chemotherapy was tolerated. The patient has shown no signs of recurrence or organ dysfunction, including the kidneys as of 26 months postoperatively. At present, childbearing is not desired by the patient, and they have no plan to use conserved oocytes.

## Discussion

Previously reported cases of adrenal ESFT with tumor thrombus in the inferior vena cava are summarized in Table 1 [3–8]. Primary adrenal ESFT most commonly occurs in young adults, with a median age of 26 years (range 20–34 years). No sex difference has been noted. The most typical presenting complaint for patients with ESFT of the adrenal gland is abdominal pain. Median tumor diameter is 14.9 cm (range 11.3–22 cm), including the present case. Recently, ESFT including ES, extraskeletal ES, primitive neuroectodermal tumor, and Askin tumor has been recognized as originating from the primitive neural tube, with a common translocation of the *EWSR1* gene at chromosome 22q12. Approximately 85% of ESFT show translocation with *FLI-1*, and the *EWSR1*/*FLI-1* chimeric fusion gene resulting from t(11:22)(q24:q12) may lead to tumorigenesis. Ten percent of cases involve translocations with ETS-related gene (*ERG*) by t(16;21)(p11;q22), and translocations with ETS variant transcription factor 1 (*ETV1*), *ETV4* and fifth Ewing’s variant (*FEV*) have been reported rarely, each with frequencies less than 0.1% [9]. Immunostaining with anti-CD99 and FLI-1 antibody, and FISH assay to confirm fusion genes, are useful investigations to reach the final diagnosis of ESFT.

**Table 1:** Nine cases of Ewing’s sarcoma originating from the adrenal gland with inferior vena cava thrombosis

No	Report year	References	Age (years)	Sex	Laterality	Tumor diameter (cm)	Initial infiltration/metastasis	Surgical therapy	Chemotherapy	Prognosis
1	2006	Kim *et al.*	25	Female	Left	15.2	IVC + RA/lung	NR	NR	NR
2	2010	Zhang * et al.*	30	Male	Right	12	IVC	Adr + Neph (incomplete)	NR	Dead (POM 8)
3	2010	Zhang * et al.*	22	Male	Left	17	IVC	Adr + Neph + Spl + IVCt	+	Alive with local rec. (POM 1)
4	2012	Saboo * et al.*	26	Female	Left	NR	Kidney + spleen + IVC	Adr + Neph + Spl + IVCt	+	Alive
5	2013	Abi-Raad * et al.*	26	Female	Left	11.3	IVC	Adr + Neph + Spl + IVCt	+^a^	Alive (POM 8)
6	2019	Christopher * et al.*	34	Male	Right	14.5	IVC	Adr + IVCt	+	Alive (POM 3)
7	2022	Ji-Lian * et al.*	20	Female	Right	22	IVC + RA/liver	Adr + IVCt + RAt	+^b^	Alive (POM 20)
8	2022	Present case	22	Female	Left	20	IVC + RA	Adr + IVCt + RAt + Pt	+^c^	Alive (POM 25)

*IVC* inferior vena cava *RA* right atrium, *IVCt* inferior vena cava thrombectomy, *Adr* adrenalectomy, *Neph* nephrectomy, *Spl* splenectomy, *RAt* right atrium thrombectomy, *Pt* pulmonary thrombectomy, *NR* not recorded, *POM* postoperative month, *rec* recurrence

^a^Vincristine/doxorubicin/cyclophosphamide + ifosfamide/etoposide 10 cycles

^b^Ifosfamide/etoposide 3 cycles + gemcitabine/paclitaxel 6 cycles

^c^Vincristine/doxorubicin/cyclophosphamide + ifosfamide/etoposide 7 cycles

Radiological imaging, including CT, MRI, and FDG-PET, contributes to the initial detection and determination of the extent of tumors and distant metastases [7]. In our case, CT and MRI also contributed to diagnosis of the origin, detailed surgical planning, and detection of tumor emboli in the pulmonary artery after the initial surgery.

The standard therapy for adrenal ESFT is complete resection and combination chemotherapy, as in this case using VDC-IE [10]. The 5- and 10-year survival rates of patients with ESFT are 69% and 62%, respectively, in patients with completely resected tumors for localized ESFT [11]. In our review of the literature, complete resection resulted in good prognosis for patients with adrenal ESFT, even with direct infiltration to the kidney, spleen, inferior vena cava, and right atrium, or even pulmonary tumor emboli from surgical maneuvers, as in the present case. Incomplete resection might be a factor associated with poor prognosis (Table 1). The standard chemotherapy for patients with adrenal ESFT is according to the traditional therapy for ES. No consensus has been reached regarding the priority of surgery or chemotherapy for primary localized adrenal ESFT. In our case, surgery was prioritized because of the symptomatic disease and tumor thrombus reaching the right atrium. Complete wide excision is necessary for patients with localized ESFT. Subsequent external irradiation therapy is recommended if complete tumor excision is not achieved, or if the patient did not show good response to prior chemotherapy [12].

## Conclusion

We encountered a rare case of primary adrenal EES with tumor thrombus in the inferior vena cava. The combination of complete tumor excision and combination chemotherapy may provide good prognosis for patients with localized ESFT.

## Data Availability

Not applicable.

## Abbreviations

ES: Ewing’s sarcoma
EES: Extraskeletal Ewing’s sarcoma
ESFT: Ewing’s sarcoma family of tumors
CT: Computed tomography
MRI: Magnetic resonance imaging
DWI: Diffusion-weighted imaging
FDG-PET: Fluorodeoxyglucose-positron emission tomography
SUV: Standardized uptake value
HE: Hematoxylin and eosin
CD: Cluster of differentiation
FLI-1: Friend leukemia integration 1 transcription factor
LCA: Leukocyte common antigen
FISH: Fluorescence *in situ* hybridization
EWSR1: Ewing’s sarcoma breakpoint region 1
VDC-IE: Vincristine, doxorubicin, cyclophosphamide-ifosfamide, etoposide
ERG: ETS-related gene
ETV1: ETS variant transcription factor 1
FEV: Fifth Ewing’s variant

## References

[1] Angervall L, Enzinger FM (1975). Extraskeletal neoplasm resembling Ewing’s sarcoma. Cancer.

[2] Machado I, Noguera R, Pellin A, Lopez-Guerrero JA, Piqueras M, Navarro S, Llombart-Bosch A (2009). Molecular diagnosis of Ewing’s sarcoma family of tumors: a comparative analysis of 560 cases with FISH and RT-PCR. Diagn Mol Pathol.

[3] Kim MS, Kim B, Park CS, Song SY, Lee EJ, Park NH, Kim HS, Kim SH, Cho KS (2006). Radiologic findings of peripheral primitive neuroectodermal tumor arising in the retroperitoneum. Am J Roentgenol.

[4] Zhang Y, Li H (2010). Primitive neuroectodermal tumors of adrenal gland. Jpn J Clin Oncol.

[5] Saboo SS, Krajewski KM, Jagannathan JP, Ramaiya N (2012). IVC tumor thrombus: an advanced case of rare extraosseous Ewing sarcoma of the adrenal gland. Urology.

[6] Abi-Raad R, Manetti GJ, Colberg JW, Hornick JL, Shah JG, Prasad ML (2013). Ewing sarcoma/primitive neuroectodermal tumor arising in the adrenal gland. Pathol Int.

[7] Ibabao C, Tsetse C, Sheth Y, Maitland C, Mohammed M (2019). Primary Ewing sarcoma of the adrenal gland: a rare cause of abdominal mass. Radiol Case Rep.

[8] Wang JL, Xu CY, Geng CJ, Liu L, Zhang MZ, Wang H, Xiao RT, Liu L, Zhang G, Ni C, Guo XY (2022). Anesthesia and perioperative management for giant adrenal Ewing’s sarcoma with inferior vena cava and right atrium tumor thrombus: a case report. World J Clin Cases.

[9] Riggi N, Stamenkovic I (2007). The biology of Ewing’s sarcoma. Cancer Lett.

[10] Grier HE, Krailo MD, Tarbell NJ, Link MP, Fryer CJ, Pritchard DJ, Gebhardt MC, Dickman PS, Perlman EJ, Meyers PA, Donaldson SS, Moore S, Rausen AR, Vietti TJ, Miser JS (2003). Addition of ifosfamide and etoposide to standard chemotherapy for Ewing’s sarcoma and primitive neuroectodermal tumor of bone. N Engl J Med.

[11] Rud NP, Reiman HM, Pritchard DJ, Frassica FJ, Smithson WA (1989). Extraosseous Ewing’s sarcoma: a study of 42 cases. Cancer.

[12] Schuck A, Ahrens S, Paulussen M, Kuhlen M, Könemann S, Rübe C, Winkelmann W, Kotz R, Dunst J, Willich N, Jürgens H (2003). Local therapy in localized Ewing tumors: result of 1058 patients treated in the CESS 81, CESS 86, and EICESS 92 trials. Int J Radiat Oncol Biol Phys.

